# Molecular mechanisms of telomere biology disorders

**DOI:** 10.1074/jbc.REV120.014017

**Published:** 2020-11-22

**Authors:** Sherilyn Grill, Jayakrishnan Nandakumar

**Affiliations:** Department of Molecular, Cellular, and Developmental Biology, University of Michigan, Ann Arbor, Michigan, USA

**Keywords:** telomere, telomerase, telomeropathies, telomerase assembly, telomerase recruitment, telomere shortening, dyskeratosis congenita, AA, aplastic anemia, BMF, bone marrow failure, CAB, Cajal body, CP, Coats plus, ds, double-stranded, DC, dyskeratosis congenita, HH, Hoyeraal–Hreidarsson, ICF, immunodeficiency, centromeric instability, and facial anomalies, IFD, insertion in fingers domain, IPF, idiopathic pulmonary fibrosis, NEXT, nuclear exosome targeting complex, PARN, poly(A)-specific ribonuclease, PCNA, proliferating cell nuclear antigen, PK/T, pseudoknot/template, PUA, pseudouridine and archeosine transglycosylase, PF, pulmonary fibrosis, RNP, ribonucleoprotein, ss, single-stranded, TBDs, telomere biology disorders, TEN, telomerase essential N-terminal domain, TERT, telomerase reverse transcriptase, TR, telomerase RNA, TERRA, telomeric repeat-containing RNA, TRBD, TERT RNA-binding domain

## Abstract

Genetic mutations that affect telomerase function or telomere maintenance result in a variety of diseases collectively called telomeropathies. This wide spectrum of disorders, which include dyskeratosis congenita, pulmonary fibrosis, and aplastic anemia, is characterized by severely short telomeres, often resulting in hematopoietic stem cell failure in the most severe cases. Recent work has focused on understanding the molecular basis of these diseases. Mutations in the catalytic TERT and TR subunits of telomerase compromise activity, while others, such as those found in the telomeric protein TPP1, reduce the recruitment of telomerase to the telomere. Mutant telomerase-associated proteins TCAB1 and dyskerin and the telomerase RNA maturation component poly(A)-specific ribonuclease affect the maturation and stability of telomerase. In contrast, disease-associated mutations in either CTC1 or RTEL1 are more broadly associated with telomere replication defects. Yet even with the recent surge in studies decoding the mechanisms underlying these diseases, a significant proportion of dyskeratosis congenita mutations remain uncharacterized or poorly understood. Here we review the current understanding of the molecular basis of telomeropathies and highlight experimental data that illustrate how genetic mutations drive telomere shortening and dysfunction in these patients. This review connects insights from both clinical and molecular studies to create a comprehensive view of the underlying mechanisms that drive these diseases. Through this, we emphasize recent advances in therapeutics and pinpoint disease-associated variants that remain poorly defined in their mechanism of action. Finally, we suggest future avenues of research that will deepen our understanding of telomere biology and telomere-related disease.

Telomeres are specialized nucleoprotein complexes that cap the ends of linear chromosomes. They consist of a repetitive DNA component (GGTTAG in humans) and a six-protein complex component, called shelterin, that binds to the telomeric DNA. Human telomeric DNA ranges from 10 to 15 kb of double-stranded (ds) telomeric repeats that terminate in a single-stranded (ss) 3’ G-rich overhang (1). Telomere shortening serves as a clock for the replicative lifespan of cells. For each cell division, telomeric DNA shortens by 50 to 200 bp owing in part to incomplete replication of the lagging strand by replicative DNA polymerases. As a result, continued cell division causes telomeres to reach a critically short length that triggers cellular senescence, limiting a cell’s capacity to proliferate. Cells requiring high proliferative potential, such as stem cells, solve this problem with the enzyme telomerase.

Telomerase is a specialized ribonucleoprotein (RNP) enzyme that adds telomeric DNA repeats to counteract the loss of telomeres that occurs during every replication cycle. Telomerase is made up of an RNA subunit TR (telomerase RNA) and a protein subunit TERT (telomerase reverse transcriptase), which together form the catalytic core of the holoenzyme (2, 3, 4, 5). TR provides the template for new telomere repeat synthesis, while TERT acts as a specialized reverse transcriptase that extends chromosome ends in a TR-template–dependent manner with new telomeric repeats.

Telomerase is activated in continually dividing cells to solve the end replication problem; however, unchecked telomerase activity in somatic cells can be tumorigenic. Indeed, telomerase is upregulated in over 90% of all cancers (6). As a result, telomerase expression is repressed in somatic cells upon differentiation, preventing unwanted telomere elongation that can lead to replicative immortality. Exogenous expression of telomerase in somatic cells rescues telomere lengthening, bypassing senescence and allowing for continuous cell division (7). However, telomerase expression does not transform cells, suggesting that it is cancer-promoting rather than fully oncogenic (8). In contrast, loss of telomerase in the continually dividing cells of the germline leads to progressive telomere shortening over many generations that eventually culminates in senescence (9, 10).

Human germline mutations in genes that are involved in telomerase function or telomere maintenance result in a range of clinical conditions that are collectively termed telomeropathies. These telomere biology disorders (TBDs) often stem from severely short or dysfunctional telomeres. The most severe TBDs eventually lead to hematopoietic stem cell failure, the major cause of morbidity and mortality in these patients (11, 12). The clinical manifestations of TBDs are thought to arise from severely short telomeres that limit replicative capacity, leading to the loss of critical stem cell pools. TBD-causative mutations have been mapped to several genes that span numerous macromolecular complexes and pathways involved in telomere maintenance (Fig. 1). Specifically, mutations in 14 different genes have been identified to date in patients with TBDs (Table 1). Of these 14 genes, TERT and TR, as well as poly(A)-specific ribonuclease (PARN), Dyskerin, NHP2, NOP10, NAF1, and TCAB1, are important for processing and assembly of the telomerase RNP (Fig. 1).

**Figure 1:**
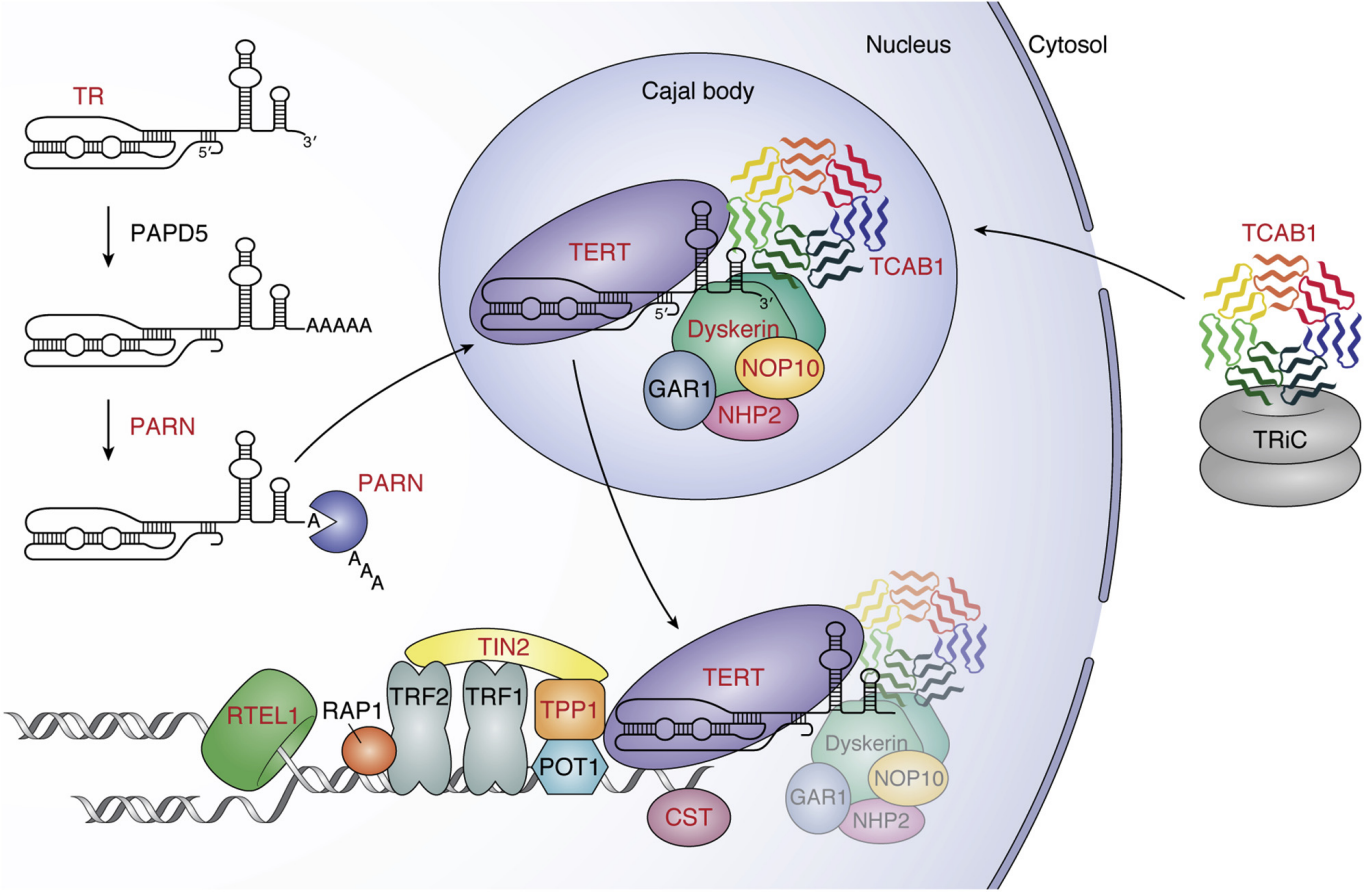
**Model for telomerase biogenesis and telomere maintenance**. TBD-associated proteins and RNA (TR) are labeled in *red*. TCAB1 (*multicolored*) is shown in the cytosol bound to TRiC (*gray*) before localizing to Cajal bodies. TR (*black*) is processed by PAPD5 and PARN (*navy blue*) before localizing in Cajal bodies. The telomerase holoenzyme (TERT [*purple*], TR, dyskerin [*green*], NHP2 [*pink*], NOP10 [*lightorange*], GAR1 [*gray*], and TCAB1) is assembled in Cajal bodies before being recruited to telomeres by the shelterin protein TPP1 (*orange*). For simplicity, the telomerase holoenzyme is shown with only one copy of NOP10, NHP2, or GAR1. TIN2 (*yellow*) is bound to shelterin proteins TPP1, TRF1, and TRF2. The CST complex (CTC1, STN1, TEN1; *mauve*) is depicted at newly synthesized ss telomeric DNA and RTEL1 (*forest green*) is shown unwinding DNA at a replication fork.

**Table 1:** TBD-associated genes

Gene	Encoded protein	Disease association	Inheritance
*TERT*	TERT	HH, DC, PF, AA	Autosomal dominant or autosomal recessive
*TR* or *TERC*	–	HH, DC, PF, AA	Autosomal dominant
*DKC1*	Dyskerin	HH, DC, PF	X-linked
*NOP10*	NOP10	DC	Autosomal recessive
*NHP2*	NHP2	DC	Autosomal recessive
*NAF1*	NAF1	PF	Autosomal dominant
*PARN*	PARN	HH, DC, PF	Autosomal dominant or autosomal recessive
*ZCCHC8*	ZCCHC8	PF	Autosomal dominant
*WRAP53*	TCAB1	HH, DC	Autosomal recessive
*ACD*	TPP1	HH, AA	Autosomal dominant or autosomal recessive
*TINF2*	TIN2	HH, DC, PF, RS	Majority *de novo*, autosomal dominant
*RTEL1*	RTEL1	HH, DC, PF, AA	Autosomal dominant or autosomal recessive
*CTC1*	CTC1	DC, CP	Autosomal recessive
*STN1*	STN1	CP	Autosomal recessive

Another hotspot for TBD mutations is the shelterin complex (Fig. 1). Shelterin specifically binds to telomeric DNA to protect chromosome ends from eliciting a DNA damage response. Within shelterin, TRF1 and TRF2 (telomere repeat binding factor 1 and 2) bind the telomeric dsDNA while POT1 (protection of telomeres 1) binds to the terminal ss overhang. Rap1 associates with TRF2. TPP1 interacts with POT1 to increase POT1’s affinity for ssDNA, and TIN2 interacts with TPP1, TRF1, and TRF2 (1). While TIN2 (human gene name: *TINF2*) is frequently mutated in patients suffering from the most severe TBDs, multiple such mutations have also been described in TPP1 (human gene name: *ACD*). In addition to mutations that affect telomerase or the shelterin complex, mutations that prevent proper telomere replication are also implicated in TBDs. *CTC1* and *RTEL1* are two such genes that are associated with TBDs that facilitate proper telomere replication (Fig. 1). In this review, we discuss each of these known genes that are associated with TBDs and review the underlying molecular mechanisms that drive telomere-related disease.

## Telomere biology disorders

The first telomere biology disorder was described in 1998 with the identification of patients suffering from a rare bone marrow failure (BMF) syndrome. These patients had germline mutations in the gene *DKC1*, which encodes the protein dyskerin. They suffered from dyskeratosis congenita (DC), a prominent TBD that is characterized by a diagnostic triad of abnormal skin pigmentation, dysplastic nails, and oral leukoplakia as well as an increased risk for BMF (13, 14, 15). These clinical features are thought to develop owing to a loss in replicative capacity resulting from severely short telomeres, often in the less than first percentile for their age. Thus, it is not surprising that tissues with high cellular turnover, such as dermal and ectodermal stem cells, reflect the classical symptoms of DC (16).

DC symptoms often appear in early childhood, with BMF developing by age 30 in the vast majority of DC patients. However, disease severity often correlates with telomere length (11). Germline mutations that impair telomere length maintenance result in the progressive shortening of telomeres in successive generations. This phenomenon, known as genetic anticipation, has been observed in mouse models lacking telomerase (9, 10). In some families affected by TBDs, disease severity increases with successive generations, suggesting that progressive telomere shortening results in a progressively earlier age of disease onset. This disease anticipation has been reported in families with TERT, TR, and TIN2 pathogenic mutations (15). However, there can be considerable variation even between patients in the same family, in both the severity of the symptoms and the age of onset.

Two severe forms of DC, Hoyeraal–Hreidarsson (HH) and Revesz syndromes, also appear in early childhood or infancy. In addition to the classical symptoms of DC, HH patients often suffer from additional neurological conditions, such as cerebellar hypoplasia and severe immunodeficiency (17). The defining feature of Revesz syndrome is the presence of bilateral exudative retinopathy, although intracerebral calcifications have also been noted frequently (16). Like Revesz syndrome, the TBD Coats plus (CP) syndrome is also characterized by bilateral exudative retinopathy and other DC-related symptoms. However, CP patients are also at a higher risk for life-threatening gastrointestinal bleeding due to vascular ectasias (15). Unlike DC patients, the vast majority of CP patients have mutations in CTC1. CP mutations have also been described in the CTC1-binding partner STN1 and the shelterin protein POT1 (18, 19).

Aplastic anemia (AA) is a common complication in DC patients. However, it was later characterized as an independent TBD when a subset of patients suffering from AA were identified with mutations in some of the same genes that underlie DC. These genes were also found mutated in patients suffering from idiopathic pulmonary fibrosis (IPF) and myelogenous leukemia, further expanding the definition of TBDs (20). Interestingly, the turnover rate for adult lung tissue is relatively low, yet murine studies suggest that telomere dysfunction can still drive alveolar epithelial stem cell failure (21, 22). While stem cell failure is a common characteristic of TBDs, it is an area of active research as to why certain stem cell pools are more acutely or severely affected by telomere dysfunction. In this regard, it has been suggested that tissues that undergo persistent damage over time, such as the lung and liver, may be more susceptible. The lack of a mouse model that effectively recapitulates these human TBDs has prevented a more complete understanding of the organismal impact of many of these mutations. However, despite this, some progress has been made at developing therapeutic strategies for the treatment of TBDs. For a more thorough discussion of the existing models and therapeutics, we point the reader to this review (23).

While many of these TBDs are associated with genes that are directly involved in telomere or telomerase function, not all diseases linked to telomere disfunction are so clearly connected to telomere-related genes. Immunodeficiency, centromeric instability, and facial anomalies (ICF) syndrome is one such example of a rare autosomal recessive disease caused by mutations in the DNA methyltransferase DNMT3B. ICF syndrome patients have reduced DNA methylation at various regions in the genome that has been linked to an increase in telomeric repeat-containing RNA (TERRA). This increase in TERRA may contribute to the telomere shortening seen in some ICF syndrome patients (24, 25).

## Telomerase maturation and assembly

The mature human telomerase holoenzyme contains a variety of subunits that can be distributed into two classes: stably associated factors and transiently associated factors. Within the catalytic core of telomerase, TR acts as a scaffold for many of these stably associated holoenzyme factors. TR contains an H/ACA domain that is bound by dyskerin, a member of the H/ACA ribonucleoprotein complex. Along with H/ACA RNP proteins NOP10, NHP2, and GAR1, dyskerin acts as a member of the telomerase holoenzyme to stabilize TR. In addition to the H/ACA domain, TR also contains a CAB motif, which is bound by the protein TCAB1. Together, TERT, TR, the H/ACA RNP complex proteins (dyskerin, NHP2, NOP10, and GAR1), and TCAB1 make up the telomerase holoenzyme (Fig. 1) (26, 27).

**Figure 2:**
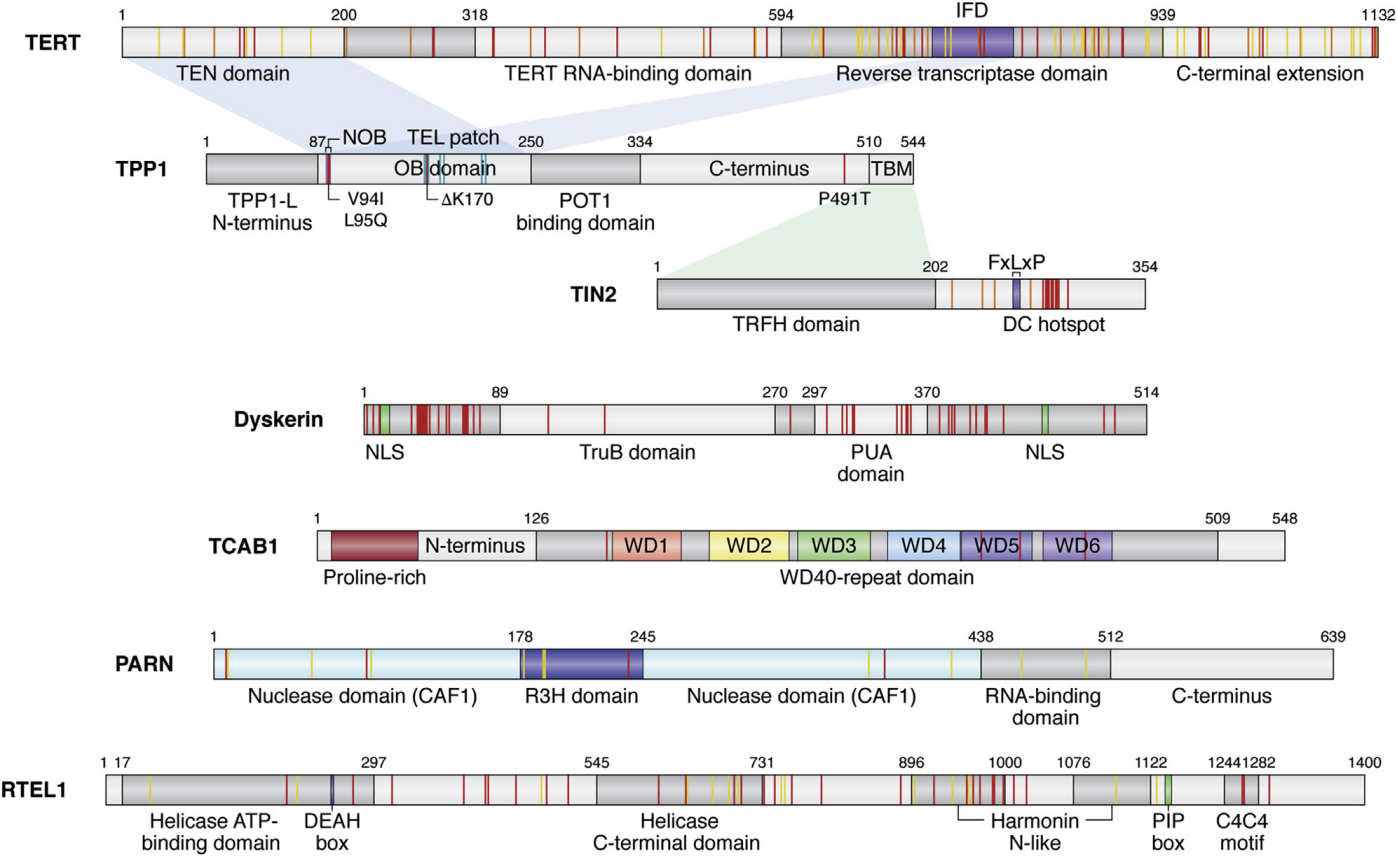
**Domain organization of TERT, TPP1, TIN2, TCAB1, PARN, and RTEL1**. Selected DC and HH mutations are shown in *red*, AA mutations are shown in *orange*, and PF mutations are shown in *yellow*. Amino acid numbering above the schematics indicates domain boundaries. Shading connecting domains of one protein to another represents protein–protein interactions mediated through the specified domains. The following proteins are described: TERT (Telomerase essential N-terminal domain [TEN], insertion in fingers domain [IFD; *purple*]). TPP1 (TIN2-binding motif of TPP1 [TBM], N-terminus of the OB [NOB; *lavender*], TEL patch residues are shown in *cyan*). TIN2 (TRF1 binding motif [FxLxP; *navy blue*], DC hotspot represents a cluster of DC mutations in TIN2). Dyskerin (pseudouridine synthase catalytic domain [TruB], RNA-binding domain [PUA]). TCAB1 (each of six WD repeats is indicated in the WD40-repeat domain). PARN (R3H domain [*blue*] splits the CAF1 nuclease domain [*aqua*]). RTEL1 (DEAH box [*blue*], PCNA binding box [PIP box-*green*], metal-coordinating C4C4 motif thought to bind TRF2 [C4C4 motif]).

**Figure 3:**
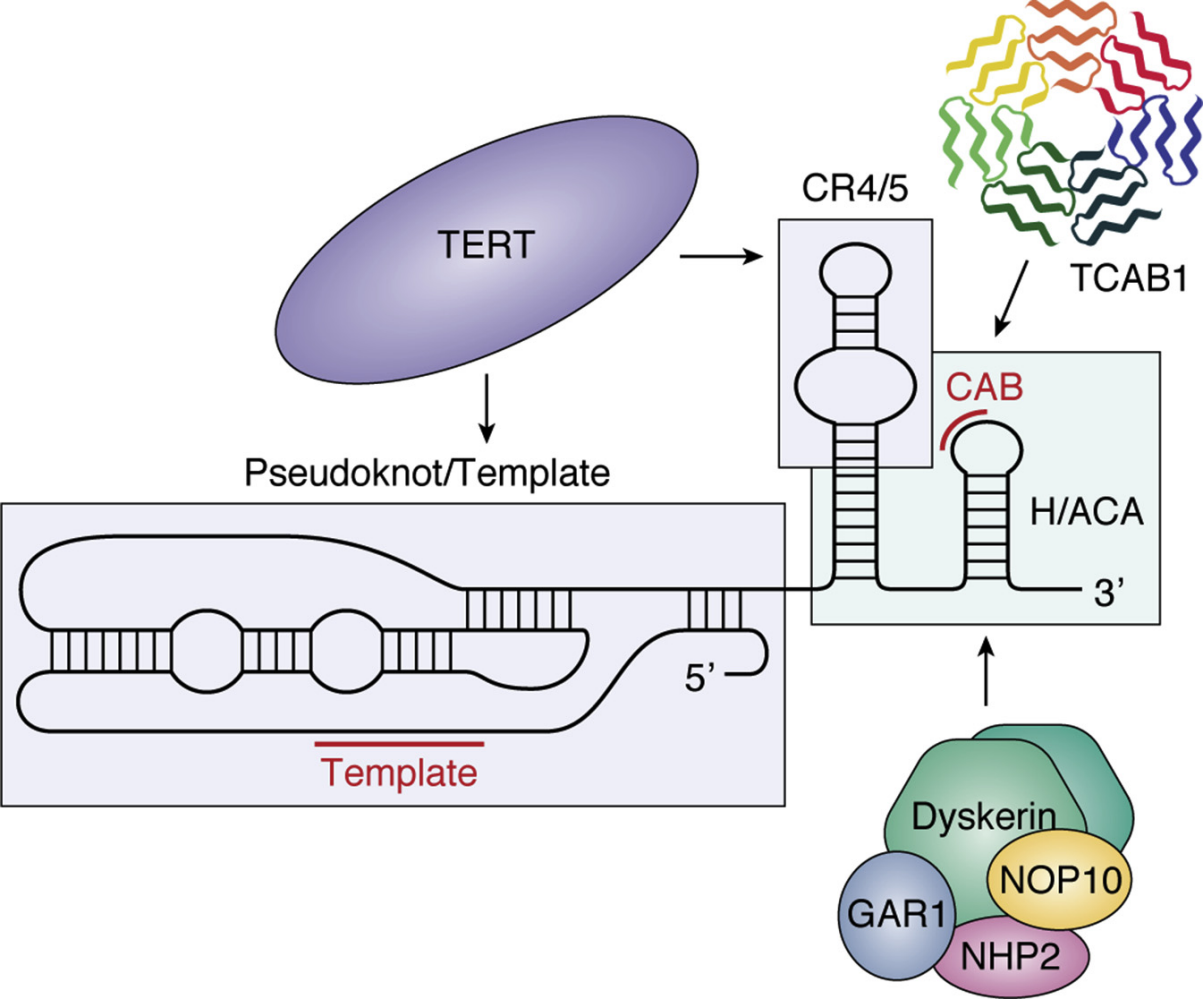
**Domain organization and interactions of TR**. A schematic of TR secondary structure divided into domains (demarcated by boxes) containing key features (CAB box and template) highlighted in *red*. Arrows point to the regions of TR that indicated telomerase RNP proteins bind to.

Many of these components are mutated in telomere biology disorders, with the majority of these mutations resulting in reduced telomerase biogenesis or function. Pathogenic mutations in the transiently associated factor NAF1, which is a component of the pre-H/ACA RNP, are also found in patients with pulmonary fibrosis (PF) (28). Within TERT, the TERT RNA-binding domain (TRBD), reverse transcriptase (RT) domain, and C-terminal extension (CTE) make up a ring-like structure, forming the catalytic reverse transcriptase (RT) core (Fig. 2) (29). The telomerase essential N-terminal domain (TEN) binds the shelterin protein TPP1, facilitating telomerase recruitment to the telomere (30, 31, 32, 33, 34, 35, 36). Not surprisingly, mutations in TERT that are associated with telomere biology disorders are varied and extensive, with all four of its critical domains affected. By comparison, TR is mutated less frequently than TERT, yet these mutations are still found throughout TR’s 451 nucleotides. Many TR mutations cluster within the pseudoknot/template domain, which contains the template sequence for reverse transcription and is critical for catalytic activity (Fig. 3) (37, 38, 39, 40, 41). The CR4/5 domain, which is the primary TERT binding region in TR, is another frequently mutated region in disease (42). TR levels depend not only on proper H/ACA RNP formation but also on the accurate processing of the TR transcript, as mutations in the poly(A)-specific ribonuclease PARN interrupt proper 3’ end processing of nascent TR, leading to a decrease in mature TR transcripts. In the next section, we discuss each of these mechanisms in detail, highlighting how pathogenic mutations in these components interfere with telomerase maturation and assembly.

### TERT

In 2005, numerous patients with DC or AA were found to harbor mutations in the *TERT* gene (43, 44, 45). Of these originally identified patients, a three-generation pedigree with autosomal dominant DC was found to have a K902N mutation in the RT region of TERT. The K902N mutation was defective in telomerase activity but did not exhibit a dominant-negative effect, suggesting that the mechanism of action was haploinsufficiency (44). Since this early report, numerous additional mutations in TERT have been reported in patients with short telomeres (Fig. 2) (46, 47, 48, 49, 50, 51). While many of these TERT mutational phenotypes are consistent with haploinsufficiency arising from reduced TERT stability, DC-associated mutants R972H and R979W within the TERT CTE domain exhibit a decrease in both telomerase activity and processivity, but not stability, compared to wild-type TERT (52, 53). Structural and biochemical analysis of the human CTE suggests that these residues extend toward the center of the TERT ring, disrupting the RNA–DNA hybrid that is essential for telomerase catalytic function (52).

The second cluster of TBD mutations lies within the insertion in fingers domain (IFD) of the TERT RT domain (54, 55). Based on homology with the *Tetrahymena thermophila* telomerase structure, the IFD contains an extended beta-sheet structure (called the IFD-TRAP; (56)) that along with the TEN domain creates a lid over the catalytic core to facilitate telomerase processivity (32, 54, 56, 57). These interactions appear to be further stabilized by the shelterin protein TPP1, which is responsible for recruiting telomerase to the telomere and stimulating telomerase processivity. IFD disease-associated mutations have been shown to inhibit TPP1-mediated telomerase activation and telomerase recruitment to the telomere, consistent with the importance of the TPP1–TEN–IFD complex for telomerase function (31, 32, 54, 57).

Although some additional pathogenic mutations in TERT have been shown to have reduced catalytic activity, surprisingly, many of them exhibit wild-type activity and processivity in direct telomerase assays (53). This inconsistency in phenotype might be explained if a modest deficit in telomerase activity is sufficient to cause telomere shortening over multiple generations or if mutations in TERT disrupt other aspects of telomerase function. One such example is the mutation of amino acid V144 in the TERT TEN domain, a variant found in patients with IPF. While this mutant shows only a modest decrease in telomerase activity compared with that in the wild-type (58), it is nonetheless defective in telomerase recruitment to telomeres (34). Additionally, with the vast array of reported mutations in TERT, it is possible that some TERT variants may not be the sole etiology of the resulting disease. Therefore, while the mechanistic underpinnings of many TERT mutations as they relate to TERT protein stability, holoenzyme assembly, or telomerase trafficking are not well investigated, the overwhelming majority of the disease-associated TERT variants are thought to function through haploinsufficiency.

### TR

The first reported TR mutations in patients with TBDs were characterized in 2001 based on their autosomal dominant mode of inheritance (59). Three separate families suffering from DC were identified, each with a unique mutation in TR. One pedigree harbored a 74 nucleotide deletion at the 3’ end of TR (Δ378–451) that resulted in the loss of the H/ACA domain (Fig. 3). The 74 bp deletion TR transcript was not detected in lymphocyte cell lines derived from the affected individuals, indicating that this transcript did not accumulate in cells. While deletion of an entire domain is a drastic mutation, telomeropathy mutations at residues 377, 448, and 450 within this deleted region have been observed subsequently (48, 50, 60), confirming the importance of nucleotides within the H/ACA domain for telomerase function. The second family was heterozygous for a single-point mutation at position 408 (C408G) within the hairpin P8 stem of the H/ACA domain (59). Biochemical experiments demonstrated that the C408G mutation results in a reduction in RNA accumulation (61, 62). Consistent with this, the recent ∼8 Å cryo-EM reconstruction of the human telomerase holoenzyme depicts dyskerin interacting with the stems of hairpins P7 and P8 of TR (26). Disruption of the TR–dyskerin interaction in the presence of these disease mutations provides a straightforward explanation for the reduction in TR stability (14, 27), pointing to haploinsufficiency as the mechanism of action for TR-destabilizing mutations (63, 64).

The third identified family contained a double-point mutation resulting in a GC to AG substitution at positions 107 and 108 within the pseudoknot/template (PK/T) region (Fig. 3) (59). In contrast to mutations in the H/ACA domain, mutation of GC107-108AG inhibited the reconstitution of active telomerase, despite wild-type levels of RNA accumulation (62). However, this disruption of enzymatic activity was not due to a decrease in TERT binding, as many of the pathogenic PK/T region variants retain the ability to interact with TERT but do not confer active telomerase (65). Instead, mutations in the PK/T region are thought to perturb the pseudoknot structure (66, 67), thereby impacting the active site configuration through a mechanism that has yet to be elucidated. Remarkably, the loss of telomerase activity in either the H/ACA domain or the PK/T region can be rescued with compensatory mutations in TR that restore the native structure (68, 69). In contrast to PK/T region mutations, aplastic anemia mutation G305A in the CR4/5 domain of TR is defective in TERT association, consistent with the essential role of the CR4/5 domain in forming the TERT–TR RNP complex (Fig. 3) (65, 70). In summary, TBD mutations in TR can reduce TR function by diminishing steady-state TR levels, disrupting the interaction with TERT, or directly impacting the catalytic function of telomerase.

### Dyskerin

*DKC1*, which encodes the protein dyskerin, was the first gene identified as responsible for DC and is known to cause the X-linked form of the disease (Table 1) (13). Dyskerin is a pseudouridine synthase that, along with GAR1, NOP10, and NHP2, comprises the H/ACA ribonucleoprotein complex involved in posttranscriptional pseudouridylation (71). Dyskerin uses small nucleolar RNAs (snoRNAs) as guides to base pair with rRNAs in the nucleolus and targets them for pseudouridylation. These snoRNAs pass through Cajal bodies for maturation before they ultimately localize to nucleoli. A subset of H/ACA guide RNAs called small Cajal body RNAs or scaRNAs remains in the Cajal body to modify small nuclear (sn)RNAs. This Cajal body retention is mediated through an interaction between TCAB1 and the scaRNA’s Cajal body (CAB) box. TR shares some of the hallmarks of scaRNAs: it contains both an H/ACA box and a CAB box and it localizes to Cajal bodies (Fig. 3). However, while TR is bound by dyskerin and the H/ACA RNP complex proteins, these interactions are not used as a guide for the pseudouridylation of snRNAs (27, 71). Instead, vertebrate TR appears to have exploited the H/ACA RNP machinery for its own stabilization. In contrast, yeast and *T. thermophila* TR do not contain an H/ACA motif, suggesting that the use of the H/ACA RNP machinery is a relatively recent adaption, consistent with the poor conservation of TR’s 3’ end and the increased complexity of mammalian TR.

Because human telomerase RNA had been shown to contain a region resembling an H/ACA domain (72), in 1999, Mitchell *et al.* (14) hypothesized that dyskerin may also interact with TR as a component of the telomerase RNP. Indeed, dyskerin was found to associate with telomerase RNA as part of the active telomerase RNP. This hallmark study revealed that primary fibroblasts from DC-affected patients were not deficient in snRNA accumulation, but instead had reduced TR levels and lower telomerase activity, resulting in shorter telomeres than cells from clinically unaffected relatives. While DC had been previously described as a BMF syndrome, this was the first study to connect DC with telomere dysfunction, paving the way for the subsequent discovery of telomeropathy mutations in several other telomerase- and telomere-associated factors. Interestingly, because dyskerin is X-linked, female *DKC1* mutation carriers present with milder clinical features of DC, possibly owing to skewed X-chromosome inactivation or germline mosaicism (73, 74).

Since the first characterized dyskerin mutations in patients with TBDs, multiple mutational hotspots have been described in the DKC1 gene. The majority of these mutations reside in two distinct portions of the protein: the N-terminus and the PUA (pseudouridine and archeosine transglycosylase) domain (Fig. 2). Dyskerin is the mammalian orthologue of Cbf5p, a *Saccharomyces cerevisiae* pseudouridine synthase that is associated with H/ACA RNAs. The PUA domain is dyskerin’s putative RNA-binding domain (71). Based on the structure of the archaeal Cbf5–NOP10–GAR1 complex, DC mutations in the PUA domain of human dyskerin are predicted to reside near the RNA–protein interface (75, 76). These mutations do not disrupt dyskerin–NOP10–NHP2 complex formation, but instead disrupt TR assembly with the H/ACA RNP (77). Accordingly, placement of the archaeal H/ACA RNP structure into the human telomerase holoenzyme cryo-EM map suggests that telomeropathy mutations within dyskerin cluster at the interface between two dyskerin molecules and the telomerase RNA (26). Consistent with defects in dyskerin–TR binding, patient cells harboring mutations in either the N-terminus or the PUA domain have reduced steady-state levels of TR and a reduction in telomerase activity that can be rescued through overexpression of wild-type TR (78, 79, 80). Taken together, pathogenic mutations in dyskerin prevent stable assembly of the H/ACA RNP with TR, thereby impairing TR stability and resulting in a reduction in telomerase activity that is causative of the telomere shortening seen in TBDs.

### NOP10, NHP2, and NAF1

Along with dyskerin, NOP10, NHP2, GAR1, and NAF1 are all members of the H/ACA RNP complex. While NAF1 is assembled as part of the pre-H/ACA RNP, it is subsequently replaced by GAR1 to form the mature H/ACA RNP complex. For a more in-depth discussion of TR assembly factors, we point the readers to reviews that describe this process in detail (27, 71). While these assembly factors are not necessary to reconstitute telomerase activity *in vitro*, they are essential for telomerase function and telomere maintenance *in vivo*. To date, mutations in NOP10, NHP2, and NAF1, as well as dyskerin, have been characterized in TBDs. Mutations in each of these factors affect TR assembly or stability through the defective formation of the telomerase RNP complex (Fig. 3). The first mutation in NOP10 was reported in 2007 when three related individuals presenting with classical DC symptoms were homozygous for the R34W mutation (81). The affected individuals had short telomeres and low TR levels, similar to what had been observed for pathogenic dyskerin mutations. NOP10 R34W did not impact pre-H/ACA RNP assembly of the NOP10–NHP2–dyskerin–NAF1 complex, but instead prevented successful association with TR (77). A year later, mutations in NHP2 were similarly found in DC patients with short telomeres and low TR levels (82). Despite this similarity with NOP10 and DKC1 mutations, NHP2 mutations instead impacted pre-H/ACA RNP assembly by preventing NHP2 binding to NOP10 (77). This not only reduced TR levels, but also the levels of all H/ACA RNAs that were tested. Notwithstanding this difference, mutations in either NOP10 or NHP2 result in a reduction of TR levels, pointing to haploinsufficiency as the mechanism of action for these TR assembly factors. Since the discovery of DC-associated mutations in NOP10 and NHP2, additional mutations in NAF1 that result in reduced TR levels have also been characterized in patients with PF (28).

### PARN

Defects in the H/ACA RNP proteins are not the only means by which TR levels are reduced in TBDs. Mutations that inactivate PARN likewise reduce TR levels and are implicated in PF, AA, and DC (Fig. 2). The first link between PARN and TBDs was made in 2015 when IPF and DC patients with short telomeres were identified to have mutations in PARN, suggesting that PARN function may be essential for telomere maintenance (83, 84, 85). Indeed, PARN-deficient patient and IPS cells displayed a decreased abundance of TR and a reduction in telomerase activity (84). In humans, TR is an independent RNA polymerase II transcript that is posttranscriptionally processed into its mature form (27). This processing pathway begins immediately following transcription with adenylation of nascent TR transcripts by the noncanonical polymerase PAPD5, which marks them for degradation (86, 87). Knockdown of PAPD5 boosts TR stability, increases telomerase activity, and causes telomere lengthening in cells (87, 88). PARN works in opposition to PAPD5, by removing the polyadenylated tails from TR transcripts, thus protecting them from degradation (Fig. 1). Disruption of PARN results in the accumulation of polyadenylated TR precursors and loss of mature TR transcripts, leading to a decrease in TR stability and reduced steady-state TR levels (84, 89, 90, 91). This decrease in TR stability compromises telomerase activity in cells with pathogenic PARN mutations, leading to the telomere shortening that is characteristic of TBDs (84). In this way, PAPD5 and PARN work in concert to tightly regulate the levels of mature TR, and thus telomerase activity, within a cell. Consistent with this, inhibition of PAPD5 rescues TR levels in PARN mutant cells, promoting telomerase activity and telomere elongation (87, 91). These studies qualified the PAPD5 enzyme as a potential therapeutic target for telomeropathies associated with reduced TR levels. The recent discovery of small molecular inhibitors of PAPD5 provides an encouraging proof of principle of this idea and highlights the clinical importance of dissecting the mechanism of action of TBDs. Two small-molecule inhibitors of PAPD5 were developed, which specifically prevent oligoadenylation of TR in cells derived from DC patients harboring pathogenic PARN mutations (92, 93). Not only did PAPD5 inhibition restore telomerase activity and telomere length *in vitro*, but continuously administered oral treatment of the inhibitor rescued wild-type telomere lengths in primary human CD34^+^ cells harboring PARN mutations in a mouse transplantation model (92). Importantly, the reduction of PAPD5 also increased TR levels in dyskerin mutant cells, suggesting that these inhibitors could be used to treat dyskerin-based TBDs (88, 92). To date, inhibitors of PAPD5 have been developed which improve the hematopoietic potential in dyskerin mutant hESCs, underscoring the potential for PAPD5 inhibition-based therapy for DC patients with reduced TR levels (94). These studies describe potential therapies that could restore telomere length in patients suffering from telomere dysfunction, a major advancement in the treatment of TBDs.

### ZCCHC8

Importantly, PARN is not the only TR-processing protein that is implicated in TBDs. ZCCHC8, a zinc finger CCHC-type domain-containing protein, is a component of the nuclear exosome targeting complex (NEXT) that was found to be mutated in a family with PF (95). The NEXT complex is responsible for the degradation of a subset of RNAs through the recruitment of the exosome to its RNA substrates (96). Using genome-wide linkage analysis, Gable *et al.* identified a loss-of-function mutation in ZCCHC8 in a proband and his two children, each of which had a 50% reduction in TR levels compared with those in healthy controls and telomere lengths below the first percentile for their age. Knockout of ZCCHC8 in HCT116 cells leads to a decrease in the fraction of mature TR and an increase in the genomically extended forms, resulting in a decrease in telomerase activity. This mirrored observations in primary fibroblasts from the proband, where extended TR forms accumulated at the expense of mature TR transcripts. Interestingly, ZCCHC8-null mice developed severe neurodevelopmental defects and exhibited 3’ end misprocessing of other low-abundance RNAs, such as cilia-encoding RNAs. How ZCCHC8 selectively targets TR and other low-abundance RNAs to the nuclear RNA exosome remains to be determined.

### TCAB1

In addition to the H/ACA RNP proteins and the TR maturation factors, additional components are required for the successful accumulation of telomerase in Cajal bodies. Specifically, the protein TCAB1 (alternative names: WRAP53 and WDR79; gene name: *WRAP53*) is essential for telomerase trafficking to Cajal bodies (97, 98). TCAB1 interacts with the CAB box on TR, helping localize telomerase in Cajal bodies and traffic it to telomeres (Fig. 3). Knockdown of TCAB1 results in the mislocalization of TR to the nucleolus and loss of telomerase at telomeres (99). Mutations in TCAB1 are extremely rare, with only two unrelated patients having been identified (100, 101). Each patient had unique compound heterozygous missense mutations in TCAB1, extremely short telomeres, and the classical DC diagnostic triad. Mutant TCAB1 protein levels in patient cells were significantly reduced compared with those in wild-type protein, with a reduction in the accumulation of TCAB1 in the nucleus. Each of the four TCAB1 mutations resides in the WD40-repeat domain (Fig. 2). This domain acts as the substrate for the chaperone TRiC, which mediates proper TCAB1 protein folding (102). Pathogenic mutations within the WD40-repeat domain disrupt this folding, causing the mutated TCAB1 protein to remain in the cytoplasm associated with TRiC. The improperly folded TCAB1 is unable to associate with TR to wild-type levels (102), resulting in mislocalization of TR to the nucleoli; however, the total amount of TR remains unchanged (100). Accordingly, cell lysates containing TCAB1 mutant proteins retained telomerase activity, despite the impaired telomerase localization *in vivo* (100). Interestingly, complete TCAB1 knockout in human cells results in a reduction of telomerase activity, suggesting that TCAB1 may support telomerase enzymatic function in addition to its essential role in telomerase trafficking (103). For pathogenic TCAB1 mutations, this is consistent with a partial loss-of-function mechanism, resulting in mislocalization of TR, impaired telomerase recruitment to the telomere, and thus telomere shortening (100).

## The telomeric shelterin complex

Both TPP1 (gene name: *ACD*) and TIN2 (gene name: *TINF2*) are members of the shelterin protein complex that binds and protects telomeric DNA from the DNA damage response machinery (Fig. 1) (104). As a member of the shelterin complex, TPP1 plays an essential role in end protection, but it also plays an essential role in end replication by recruiting telomerase to the telomere. Two isoforms of TPP1 have been reported in human cells, TPP1-L and TPP1-S, differing by only 86 amino acids at the N-terminus. TPP1-L is present in the differentiating germ cells of the testes, while TPP1-S is the predominant isoform in somatic cells (105). TPP1 is the only shelterin protein known to directly contact telomerase, as the primary partner of the TPP1 OB (oligonucleotide/oligosaccharide-binding) domain is the TERT TEN domain (30, 31, 32, 33, 34). Two regions in the OB domain of TPP1, the TEL (TPP1’s glutamate [E] and leucine [L] rich) patch and the NOB (N-terminus of the OB), are critical for recruiting telomerase to the telomere and stimulating telomerase processivity. Not surprisingly, perturbation of either of these elements has a severe impact on telomerase function and telomere length (31, 36, 106). Mutations in both the TEL patch and NOB regions of TPP1 have been observed in patients suffering from telomeropathies, further underscoring the importance of the TPP1 OB domain for telomerase function (Fig. 2) (107, 108, 109).

In contrast, TIN2 acts as a scaffold within the shelterin complex, binding TRF1 and TRF2 at the double-stranded DNA and bridging it to the single-stranded DNA binding heterodimer POT1–TPP1 (104). The N-terminal TIN2 TRFH domain interacts with the C-terminus of TPP1 (Fig. 2) to bring the POT1/TPP1 heterodimer to the telomeric DNA, and mutations that disrupt this interaction result in end deprotection (110, 111, 112). Additionally, TIN2 interacts with TRF1 through a canonical FxLxP motif in the C-terminal half of the protein (Fig. 2) (110, 113). Immediately C-terminal to the FxLxP motif is a highly conserved region that is a hotspot for TBD-associated mutations; however, the structure and function of this region are currently not known (Fig. 2). Multiple isoforms of TIN2 have been identified in human cells, but the specific functions of each of these isoforms are still an area of active research (114, 115, 116). However, both the long (TIN2-L) and short (TIN2-S) isoforms of TIN2 share each of these shelterin interaction domains, with TIN2-L encompassing all 354 amino acids of TIN2-S as well as an additional 97 amino acids at its C-terminus (114).

TIN2 may also play a role in stimulating telomerase, as the TIN2–TPP1–POT1 complex stimulates telomerase processivity better than POT1–TPP1. But in contrast to TPP1, TIN2 has not been implicated in directly binding telomerase (115, 117). While TBD mutations in TPP1 result in a reduction in telomerase recruitment to the telomere and telomerase processivity *in vitro*, the mechanism of action for telomere shortening in TIN2-based TBDs appears to be less straightforward. Neither TPP1 TBD mutations nor TIN2 TBD mutations impact end protection, suggesting that mutations that elicit telomere deprotection may not be viable. Here we discuss these mechanisms in detail.

### TPP1

In 2014, the first reported cases of TPP1 mutations in TBDs were characterized. A heterozygous single amino acid deletion (ΔK170) near the TPP1 TEL patch was identified in patients from two unrelated families (Fig. 2) (107, 108). Affected individuals from both families had short telomeres, with one proband suffering from HH syndrome, a severe form of DC. Structural analysis of the TPP1 OB domain harboring the ΔK170 mutation suggests that while amino acid K170 might not directly contact TERT, it allows the TEL patch to adopt its native conformation to facilitate telomerase binding. Three critical TEL patch amino acids lie within a loop (E168, E169, E171) in the TPP1 OB domain, termed the TEL patch knuckle. Comparison of TPP1 OB wild-type and ΔK170 crystal structures revealed that loss of amino acid K170 disrupts the TEL patch knuckle structure, thereby displacing E168 and E169 from their native positions (118). Consistent with this, TPP1 ΔK170 is unable to recruit telomerase *in vivo* or stimulate telomerase processivity *in vitro* but is able to bind POT1 and protect telomeres (107, 108, 118). HEK 293T cells that were edited using CRISPR-Cas9 to contain one wild-type and one mutant copy of the TPP1 allele exhibited telomere loss over time, suggesting that a single copy of the ΔK170 mutation is sufficient to cause telomere shortening in human cells. In support of this haploinsufficiency model, TPP1 ΔK170 protein did not compromise the stimulation of telomerase processivity by wild-type TPP1, suggesting that this mutant TPP1 does not impose a dominant-negative effect (118). Notably, three nt deletions are uncommon in TBDs, yet the ΔK170 mutation was observed in two unrelated families. This not only supports a causal role for K170 in telomerase function but also suggests that the K170 codon may be a hotspot for deletion. It is intriguing to speculate that the three identical glutamate codons that surround K170 might increase the propensity for codon skipping by DNA polymerase.

Characterization of the ΔK170 mutation was the first reported link between the TPP1 protein and telomere disease. Since then, however, additional homozygous TPP1 mutations in the NOB region (V94I, L95Q) have been identified in patients with DC-like features. Patient cells harboring disease-associated NOB mutations exhibited reduced telomerase activity, and both TPP1_V94I_ and TPP1_L95Q_ were unable to pull down TERT at wild-type levels (109), consistent with prior mutagenesis studies conducted in this region (106). While the identification of two separate stretches within the primary structure of the TPP1 OB domain was unexpected, it raises interesting questions about the three-dimensional presentation of these regions as well as the nature of the TERT surface at the TERT–TPP1 interface.

### TIN2

Unlike TPP1, there is little consensus around the molecular mechanisms underlying TIN2 pathogenesis in TBDs. While the majority of TBD mutations are inherited, most pathogenic TIN2 mutations are heterozygous and arise *de novo* (16, 119). It is possible that given the critical role of TIN2, many TIN2-based TBD germline mutations are lethal during adolescent development and thus are unlikely to be passed on to other generations. These mutations often result in severe telomere shortening with an early onset of the disease. TIN2 mutations range from missense mutations to nonsense mutations, but regardless of the type, all pathogenic TIN2 mutations cluster within a DC hotspot in the protein primary structure (Fig. 2). Within that cluster, missense mutations that affect amino acids 280 to 284 (of both isoforms) are the most common, with the first DC patients being identified in 2008 with substitutions at K280 and R282 (119, 120). These patients had shorter telomeres than any other DC subtype that had been reported; however, unlike previously characterized TR and dyskerin mutations, their TR levels were unaffected (119).

The DC hotspot resides in a region of TIN2 that is shared between both the TIN2-S and TIN2-L isoforms and is outside of the TPP1, TRF1, and TRF2 binding domains. Consistent with this, TIN2 DC mutations do not decrease TIN2-S binding to shelterin partners TPP1, TRF1, or TRF2 (121, 122, 123, 124); however, one report has suggested that these mutations may decrease TRF1 binding specifically to the TIN2-L isoform (116).

A major discrepancy surrounding TIN2 pathogenesis is whether these mutations are acting through a telomerase-dependent or telomerase-independent mechanism. In 2011, Yang *et al.* (124) used human cancer cell lines to test the effect of TIN2 DC mutations on telomere shortening and telomerase function. They found that overexpression of TIN2 TBD variants led to telomere shortening in cells but had no effect on telomerase activity *in vitro*. However, TIN2 variants pulled down less TR and telomerase activity than wild-type TIN2. In agreement with this, in 2015, Frank *et al.* (122) edited human cancer cells to create heterozygous TIN2_R282H_ clones, which exhibited telomere shortening without perturbing shelterin. TIN2_R282H_ clones had reduced telomerase recruitment to the telomere, thus impairing telomere extension. However, no direct interaction between TIN2 and telomerase has been observed. Because TPP1 is both necessary and sufficient for telomerase recruitment (34), it is interesting to envision how these TIN2 variants are impacting telomerase recruitment despite having no obvious defect in TPP1 binding or shelterin localization at telomeres.

Others have described TIN2 pathogenesis as telomerase-independent. Canudas *et al.* (125) found that DC-associated mutations in TIN2 impaired sister telomere cohesion by preventing a TIN2-HP1γ (heterochromatin protein 1γ) interaction in human cells. Because previous studies had focused on understanding the effects of these mutations in cell culture, Frescas *et al.* (121) asked what the organismal effects of TIN2 TBD mutations were by generating mice harboring the equivalent of the human TIN2 K280E mutation (mTIN2_K267E_). Crosses between TIN2^+/K267E^ mice did not produce any homozygous DC mice, suggesting that the TIN2 DC allele is lethal in this context. This is striking as the homozygous deletion of mTR yields viable mice for multiple generations, suggesting that the TIN2 DC phenotype is more severe than that of the complete loss of telomerase (9). Importantly, mTR knockout mice harboring the heterozygous TIN2 DC mutation had significantly shorter bone marrow telomeres than mTR knockout littermates with two copies of wild-type TIN2, supporting, at least in part, a telomerase-independent mechanism of telomere shortening (121).

The narrative surrounding TIN2 TBD mutations becomes even more intriguing in light of the genetic peculiarities observed in certain patients. While the vast majority of patients with TIN2 mutations have a severe form of DC that results in BMF at a young age, not all patients with these mutations suffer from such a severe phenotype. Specifically, two unrelated adults with IPF, ages 43 and 49 years old, were found to have mutations in TIN2 and extremely short telomeres (126, 127). However, these patients did not present with severe hematological symptoms or BMF. The 49-year-old patient had a germline mutation in TIN2 at amino acid 284, a residue that is frequently mutated in TIN2-based DC, as well as a second, acquired 15 bp deletion on the same allele (126). The deletion spanned the intron 5-exon 6 boundary and is expected to result in a functionally null protein. Remarkably, tissue extracted from the lungs of the patient was largely devoid of the deletion mutation (8% of clones, 40% contained the T284R), while the majority of clones derived from the blood carried the deletion (61% of clones). The authors hypothesized that this mosaicism may be the result of functional reversion, as the DC-associated T284 mutation is effectively silenced by the deletion mutation on the same allele. This functional reversion may have played a protective role against the BMF that is commonly associated with mutation of TIN2, suggesting that the T284R mutation acts through a dominant-negative mechanism that can be suppressed by the inactivation of the mutant allele. Interestingly, this is not the first instance that a reversion has been proposed in TIN2-based TBDs. It was suggested that the second adult IPF patient also experienced reversion of the TIN2 mutant allele; however, biological materials were not available to test this hypothesis (127, 128).

Future studies are needed to fully elucidate the mechanism of action for telomere shortening in TIN2-based TBDs. These should include unbiased approaches that look at both telomerase-dependent and telomerase-independent mechanisms. Additionally, further investigation into the biochemical underpinnings of the potential dominant-negative nature of TIN2 in TBDs seems mechanistically intriguing.

## Telomere replication

### RTEL1

RTEL1 is an essential helicase that plays a critical role in the resolution of DNA secondary structures that arise during DNA replication, repair, and recombination. It belongs to both the DEAH subfamily of superfamily 2 helicases, which contain a helicase domain with 5’ to 3’ helicase activity, as well as the iron–sulfur (Fe-S) cluster helicase family (Fig. 2) (129). Through its PIP box, RTEL1 interacts with proliferating cell nuclear antigen (PCNA) to support genome-wide DNA replication (130). Through its associations with PCNA and shelterin component TRF2, RTEL1 additionally plays a critical role in telomere replication and the resolution of t-loop structures that cap chromosome ends to protect the ss telomeric overhang. RTEL1 appears to be essential for resolving these telomeric DNA structures, as conditional knockout of *Rtel1* in MEFs results in fragile telomeres, rapid accumulation of telomere circles (t-circles), and increased telomere length heterogeneity (131). RTEL1 is recruited to telomeres by the shelterin protein TRF2 in late S phase. Loss of this interaction leads to aberrant t-loop excision, inducing t-circle formation and telomere length heterogeneity (132). Dephosphorylation of TRF2 during S-phase supports the TRF2-RTEL1 interaction, allowing RTEL1 to unwind t-loops and facilitate telomere replication. Rephosphorylation of TRF2 outside of S-phase then releases RTEL1 from telomeres (133). Interestingly, RTEL1 has also been shown to interact with TRF1, suggesting a potential additional mechanism for telomere association (134). In contrast, the PCNA–RTEL1 interaction is required to counteract G-quadruplex structures in telomeric DNA, and loss of PCNA binding leads to fragile sites at telomeres that result from telomere replication defects (130).

In 2013, the first TBDs associated with RTEL1 mutations were identified (134, 135, 136, 137, 138). RTEL1 mutations have been found in both HH patients and patients with familial pulmonary fibrosis, with mutations scattered throughout much of RTEL1’s coding region (16). Patients who develop the most phenotypically severe RTEL1-based TBDs, such as DC and HH, often have compound heterozygous or homozygous mutations, while heterozygous mutations are more frequently identified in PF patients (83, 134, 135, 137, 138). RTEL1 HH mutations are associated with very short telomeres and are often accompanied by replication defects, such as increased DNA damage and telomere fragility (135, 136, 137, 138), consistent with RTEL1’s essential role in telomere replication. TBD-associated mutations in the helicase domain and PIP box have been described, as well as many mutations in regions of the protein with unknown function. Additional functions such as a role in stabilizing telomeric G-overhangs and trafficking of pre-U2 RNA have also been described (139, 140), but future studies probing individual RTEL1 TBD-associated mutations are necessary to fully elucidate the mechanisms underlying these variants.

Many RTEL1 mutations impact some aspect of telomere replication, including HH-associated mutation R1264H, which specifically affects proper t-loop resolution. Importantly, RTEL1 R1264H has a carrier frequency of 1% in the Ashkenazi Jewish population (135, 141). R1264 resides in the C4C4 motif that is responsible for coordinating Zn^+2^ in RTEL1’s RING finger motif, a motif that has been proposed to facilitate RTEL1’s interaction with TRF2 (133, 142). In agreement with this, expression of RTEL1^R1237H^ prevents RTEL1 sequestration at telomeres in cells expressing a phospho-dead TRF2 that is deficient in RTEL1 release (133). Patient cells harboring RTEL1^R1264H^ exhibit extreme telomere length heterogeneity and a significant increase in t-circle formation, consistent with inappropriate t-loop resolution (135). Interestingly, MEFs harboring the equivalent mouse mutation (R1237H) did not exhibit signs of telomere fragility or loss of PCNA binding, despite an increase in t-circle formation, suggesting that the R1264H mutation represents a separation-of-function mutation that impacts t-loop resolution but not telomere replication.

### CTC1

CTC1 is a member of the CST complex, composed of proteins CTC1, STN1, and TEN1. Chromosome end replication not only involves the addition of G-rich telomeric repeats to the 3’ end of telomeres by telomerase, but also requires subsequent C-strand fill-in synthesis. The CST complex aids in C-strand synthesis by binding to the telomeric 3’ overhang and stimulating DNA polymerase α-primase, while at the same time inhibiting telomere elongation by blocking telomerase access to the 3’ tail and limiting telomerase stimulation by POT1–TPP1 (143, 144, 145, 146). CST has been implicated in binding to POT1–TPP1, providing a mechanism for its recruitment to chromosome ends (143, 147, 148). However, because CST also binds the G-rich ss DNA tail, like the POT1-TPP1 heterodimer, it may also compete with POT1–TPP1 for telomeric DNA binding. In addition to its telomeric functions, CST has been shown to have genome-wide functions in rescuing replication fork stalling (144, 149, 150, 151, 152, 153, 154).

CTC1 has been implicated in both traditional telomeropathies, such as DC, and CP. Both DC and CP patients with CTC1 mutations have presented with extremely short telomeres, yet interestingly not all patients with CTC1 mutations have this phenotype (155, 156, 157). The vast majority of TBD-associated CTC1 mutations are biallelic, with one allele harboring a severe frameshift mutation that results in a truncated CTC1 transcript, and the second allele harboring a potentially hypomorphic missense mutation (158, 159). Notably, many CTC1 hypomorphic point mutations exert a dominant-negative effect on telomere replication when introduced in CTC1 wild-type cells, yet in patients, these mutations are combined with loss-of-function alleles rather than wild-type.

CTC1 missense mutations can affect any of CST’s known functions, with mutations occurring in the C-terminus of CTC1 impacting CST complex formation, while mutations A227V and V259M in CTC1’s N-terminal OB domain inhibit its polα-primase interaction (158). Additional DC and CP-associated mutations have been shown to impede telomeric DNA binding, despite having no effect on CST complex formation. Notably, CP-associated mutation CTC1^L1142H^ disrupts STN1 binding, reducing CST’s interaction with DNA pol-α and telomeric ss DNA. This leads to increased telomerase recruitment to telomeres and telomere lengthening, suggesting that CTC1^L1142H^ is unable to fully repress telomerase activity. However, despite significant telomere lengthening in the short term, extensive passaging of cells harboring CTC1^L1142H^ resulted in progressive telomere shortening, presumably due to defective maintenance of the C-strand (160). The recently solved cryo-EM structure of human CST bound to ss DNA reveals the distribution of disease mutations across the multiple OB domains of the CTC1 protein (161). Curiously, while the loss of either CST complex formation or polα-primase binding prevents proper localization of CTC1 to the telomere, none of the characterized mutations abolished CST’s interaction with POT1–TPP1 (158). Nonetheless, the expression of each characterized CTC1 mutant disrupts telomere lagging strand synthesis, suggesting that all CTC1 mutations result in compromised telomeric DNA replication. While STN1 has not been described in patients suffering from HH or DC, multiple STN1 mutations have been identified in CP patients. These patients exhibit telomere replication defects and abnormal C-strand fill-in (19). Remarkably, no mutations in TEN1 have been identified in telomeropathy patients to date, despite its essential role in CST function.

## Conclusions

Despite a growing understanding of TBDs and the mechanisms that underlie them, there is no cure for these diseases today. However, with the emergence of new inhibitors against PAPD5 for those patients with PARN, dyskerin, or TR-based TBDs, there is hope that a successful treatment for a subset of TBD patients could become available in the not-so-distant future. In the less than 6 years since the identification of PARN mutations in TBDs, researchers have decoded the mechanistic details of PARN’s effect on telomerase and telomere biology and have developed inhibitors to PAPD5 that can successfully rescue telomere length in PARN- and dyskerin-deficient cells. This remarkable advancement is not only a testament to the importance of understanding disease variants at a molecular level, but also an impetus to continue probing how each of these genes functions in telomere biology. Researchers should persist in their efforts to look for new gene candidates and reevaluate known genes, as our understanding of telomere biology improves to catch up with the ∼30% of DC cases that remain uncharacterized at the genetic or biochemical level (162). Critical for this process is the use of an organismal TBD disease model that can be used to evaluate future treatments. Laboratory mice maintain significantly longer telomeres than humans, and thus they typically require many generations of successive telomere attrition before exhibiting any signs of telomere dysfunction (9, 163). Acute inactivation of *Acd*, the gene that encodes TPP1, in mouse bone marrow hematopoietic stem and progenitor cells induces hematopoietic failure and HSC loss, providing a potential model for telomere dysfunction-induced HSC failure (164). Yet unlike DC patients with the TPP1ΔK170 mutation, the equivalent mouse mutation TPP1ΔK82 did not recapitulate this hematopoietic stem cell failure in a mouse HSC transplantation model (unpublished data). Thus, current research uses transplanted human hematopoietic stem cells in immunodeficient mice as the best model for hematopoietic stem cell longevity in TBDs (92). While the use of IPS and ES cells has been invaluable for our understanding of TBDs *in vitro* (101, 165), deciphering the organismal basis for TBD disease phenotypes may be the next essential step in moving forward with potential treatments.

## Conflict of interest

The authors declare that they have no conflicts of interest with the contents of this article.

## References

[1] Palm W., de Lange T. (2008). How shelterin protects mammalian telomeres. Annu. Rev. Genet..

[2] Blackburn E.H., Collins K. (2011). Telomerase: an RNP enzyme synthesizes DNA. Cold Spring Harb. Perspect. Biol..

[3] Greider C.W., Blackburn E.H. (1987). The telomere terminal transferase of tetrahymena is a ribonucleoprotein enzyme with two kinds of primer specificity. Cell.

[4] Blackburn E.H., Greider C.W., Henderson E., Lee M.S., Shampay J., Shippen-Lentz D. (1989). Recognition and elongation of telomeres by telomerase. Genome.

[5] Lingner J., Hughes T.R., Shevchenko A., Mann M., Lundblad V., Cech T.R. (1997). Reverse transcriptase motifs in the catalytic subunit of telomerase. Science.

[6] Kim N.W., Piatyszek M.A., Prowse K.R., Harley C.B., West M.D., Ho P.L., Coviello G.M., Wright W.E., Weinrich S.L., Shay J.W. (1994). Specific association of human telomerase activity with immortal cells and cancer. Science.

[7] Bodnar A.G., Ouellette M., Frolkis M., Holt S.E., Chiu C.P., Morin G.B., Harley C.B., Shay J.W., Lichtsteiner S., Wright W.E. (1998). Extension of life-span by introduction of telomerase into normal human cells. Science.

[8] Morales C.P., Holt S.E., Ouellette M., Kaur K.J., Yan Y., Wilson K.S., White M.A., Wright W.E., Shay J.W. (1999). Absence of cancer-associated changes in human fibroblasts immortalized with telomerase. Nat. Genet..

[9] Blasco M.A., Lee H.W., Hande M.P., Samper E., Lansdorp P.M., DePinho R.A., Greider C.W. (1997). Telomere shortening and tumor formation by mouse cells lacking telomerase RNA. Cell.

[10] Lee H.W., Blasco M.A., Gottlieb G.J., Horner J.W., Greider C.W., DePinho R.A. (1998). Essential role of mouse telomerase in highly proliferative organs. Nature.

[11] Dokal I. (2011). Dyskeratosis congenita. Hematology Am. Soc. Hematol. Educ. Program.

[12] Jones M., Bisht K., Savage S.A., Nandakumar J., Keegan C.E., Maillard I. (2016). The shelterin complex and hematopoiesis. J. Clin. Invest..

[13] Heiss N.S., Knight S.W., Vulliamy T.J., Klauck S.M., Wiemann S., Mason P.J., Poustka A., Dokal I. (1998). X-linked dyskeratosis congenita is caused by mutations in a highly conserved gene with putative nucleolar functions. Nat. Genet..

[14] Mitchell J.R., Wood E., Collins K. (1999). A telomerase component is defective in the human disease dyskeratosis congenita. Nature.

[15] Niewisch M.R., Savage S.A. (2019). An update on the biology and management of dyskeratosis congenita and related telomere biology disorders. Expert Rev. Hematol..

[16] Bertuch A.A. (2016). The molecular genetics of the telomere biology disorders. RNA Biol..

[17] Ohga S., Kai T., Honda K., Nakayama H., Inamitsu T., Ueda K. (1997). What are the essential symptoms in the Hoyeraal-Hreidarsson syndrome?. Eur. J. Pediatr..

[18] Takai H., Jenkinson E., Kabir S., Babul-Hirji R., Najm-Tehrani N., Chitayat D.A., Crow Y.J., de Lange T. (2016). A POT1 mutation implicates defective telomere end fill-in and telomere truncations in coats plus. Genes Dev..

[19] Simon A.J., Lev A., Zhang Y., Weiss B., Rylova A., Eyal E., Kol N., Barel O., Cesarkas K., Soudack M., Greenberg-Kushnir N., Rhodes M., Wiest D.L., Schiby G., Barshack I. (2016). Mutations in STN1 cause coats plus syndrome and are associated with genomic and telomere defects. J. Exp. Med..

[20] Savage S.A., Bertuch A.A. (2010). The genetics and clinical manifestations of telomere biology disorders. Genet. Med..

[21] Povedano J.M., Martinez P., Flores J.M., Mulero F., Blasco M.A. (2015). Mice with pulmonary fibrosis driven by telomere dysfunction. Cell Rep..

[22] Alder J.K., Barkauskas C.E., Limjunyawong N., Stanley S.E., Kembou F., Tuder R.M., Hogan B.L., Mitzner W., Armanios M. (2015). Telomere dysfunction causes alveolar stem cell failure. Proc. Natl. Acad. Sci. U. S. A..

[23] Martínez P., Blasco M.A. (2017). Telomere-driven diseases and telomere-targeting therapies. J. Cell Biol..

[24] Sagie S., Toubiana S., Hartono S.R., Katzir H., Tzur-Gilat A., Havazelet S., Francastel C., Velasco G., Chédin F., Selig S. (2017). Telomeres in ICF syndrome cells are vulnerable to DNA damage due to elevated DNA:RNA hybrids. Nat. Commun..

[25] Toubiana S., Velasco G., Chityat A., Kaindl A.M., Hershtig N., Tzur-Gilat A., Francastel C., Selig S. (2018). Subtelomeric methylation distinguishes between subtypes of immunodeficiency, centromeric instability and facial anomalies syndrome. Hum. Mol. Genet..

[26] Nguyen T.H.D., Tam J., Wu R.A., Greber B.J., Toso D., Nogales E., Collins K. (2018). Cryo-EM structure of substrate-bound human telomerase holoenzyme. Nature.

[27] MacNeil D.E., Bensoussan H.J., Autexier C. (2016). Telomerase regulation from beginning to the end. Genes (Basel).

[28] Stanley S.E., Gable D.L., Wagner C.L., Carlile T.M., Hanumanthu V.S., Podlevsky J.D., Khalil S.E., DeZern A.E., Rojas-Duran M.F., Applegate C.D., Alder J.K., Parry E.M., Gilbert W.V., Armanios M. (2016). Loss-of-function mutations in the RNA biogenesis factor NAF1 predispose to pulmonary fibrosis-emphysema. Sci. Transl. Med..

[29] Podlevsky J.D., Chen J.J. (2012). It all comes together at the ends: telomerase structure, function, and biogenesis. Mutat. Res..

[30] Schmidt J.C., Dalby A.B., Cech T.R. (2014). Identification of human TERT elements necessary for telomerase recruitment to telomeres. Elife.

[31] Nandakumar J., Bell C.F., Weidenfeld I., Zaug A.J., Leinwand L.A., Cech T.R. (2012). The TEL patch of telomere protein TPP1 mediates telomerase recruitment and processivity. Nature.

[32] Tesmer V.M., Smith E.M., Danciu O., Padmanaban S., Nandakumar J. (2019). Combining conservation and species-specific differences to determine how human telomerase binds telomeres. Proc. Natl. Acad. Sci. U. S. A..

[33] Sexton A.N., Youmans D.T., Collins K. (2012). Specificity requirements for human telomere protein interaction with telomerase holoenzyme. J. Biol. Chem..

[34] Zhong F.L., Batista L.F., Freund A., Pech M.F., Venteicher A.S., Artandi S.E. (2012). TPP1 OB-fold domain controls telomere maintenance by recruiting telomerase to chromosome ends. Cell.

[35] Armbruster B.N., Banik S.S., Guo C., Smith A.C., Counter C.M. (2001). N-terminal domains of the human telomerase catalytic subunit required for enzyme activity *in vivo*. Mol. Cell. Biol..

[36] Nandakumar J., Cech T.R. (2013). Finding the end: recruitment of telomerase to telomeres. Nat. Rev. Mol. Cell Biol..

[37] Shefer K., Brown Y., Gorkovoy V., Nussbaum T., Ulyanov N.B., Tzfati Y. (2007). A triple helix within a pseudoknot is a conserved and essential element of telomerase RNA. Mol. Cell. Biol..

[38] Theimer C.A., Blois C.A., Feigon J. (2005). Structure of the human telomerase RNA pseudoknot reveals conserved tertiary interactions essential for function. Mol. Cell.

[39] Theimer C.A., Feigon J. (2006). Structure and function of telomerase RNA. Curr. Opin. Struct. Biol..

[40] Qiao F., Cech T.R. (2008). Triple-helix structure in telomerase RNA contributes to catalysis. Nat. Struct. Mol. Biol..

[41] Chen J.L., Greider C.W. (2003). Template boundary definition in mammalian telomerase. Genes Dev..

[42] Chen J.L., Opperman K.K., Greider C.W. (2002). A critical stem-loop structure in the CR4-CR5 domain of mammalian telomerase RNA. Nucleic Acids Res..

[43] Vulliamy T.J., Walne A., Baskaradas A., Mason P.J., Marrone A., Dokal I. (2005). Mutations in the reverse transcriptase component of telomerase (TERT) in patients with bone marrow failure. Blood Cells Mol. Dis..

[44] Armanios M., Chen J.L., Chang Y.P., Brodsky R.A., Hawkins A., Griffin C.A., Eshleman J.R., Cohen A.R., Chakravarti A., Hamosh A., Greider C.W. (2005). Haploinsufficiency of telomerase reverse transcriptase leads to anticipation in autosomal dominant dyskeratosis congenita. Proc. Natl. Acad. Sci. U. S. A..

[45] Yamaguchi H., Calado R.T., Ly H., Kajigaya S., Baerlocher G.M., Chanock S.J., Lansdorp P.M., Young N.S. (2005). Mutations in TERT, the gene for telomerase reverse transcriptase, in aplastic anemia. N. Engl. J. Med..

[46] Marrone A., Walne A., Tamary H., Masunari Y., Kirwan M., Beswick R., Vulliamy T., Dokal I. (2007). Telomerase reverse-transcriptase homozygous mutations in autosomal recessive dyskeratosis congenita and Hoyeraal-Hreidarsson syndrome. Blood.

[47] Gramatges M.M., Qi X., Sasa G.S., Chen J.J., Bertuch A.A. (2013). A homozygous telomerase T-motif variant resulting in markedly reduced repeat addition processivity in siblings with Hoyeraal Hreidarsson syndrome. Blood.

[48] Collopy L.C., Walne A.J., Cardoso S., de la Fuente J., Mohamed M., Toriello H., Tamary H., Ling A.J., Lloyd T., Kassam R., Tummala H., Vulliamy T.J., Dokal I. (2015). Triallelic and epigenetic-like inheritance in human disorders of telomerase. Blood.

[49] Xin Z.T., Beauchamp A.D., Calado R.T., Bradford J.W., Regal J.A., Shenoy A., Liang Y., Lansdorp P.M., Young N.S., Ly H. (2007). Functional characterization of natural telomerase mutations found in patients with hematologic disorders. Blood.

[50] Vulliamy T.J., Kirwan M.J., Beswick R., Hossain U., Baqai C., Ratcliffe A., Marsh J., Walne A., Dokal I. (2011). Differences in disease severity but similar telomere lengths in genetic subgroups of patients with telomerase and shelterin mutations. PLoS One.

[51] Armanios M., Blackburn E.H. (2012). The telomere syndromes. Nat. Rev. Genet..

[52] Hoffman H., Rice C., Skordalakes E. (2017). Structural analysis reveals the deleterious effects of telomerase mutations in bone marrow failure syndromes. J. Biol. Chem..

[53] Zaug A.J., Crary S.M., Jesse Fioravanti M., Campbell K., Cech T.R. (2013). Many disease-associated variants of hTERT retain high telomerase enzymatic activity. Nucleic Acids Res..

[54] Chu T.W., MacNeil D.E., Autexier C. (2016). Multiple mechanisms contribute to the cell growth defects imparted by human telomerase insertion in fingers domain mutations associated with premature aging diseases. J. Biol. Chem..

[55] Podlevsky J.D., Bley C.J., Omana R.V., Qi X., Chen J.J. (2008). The telomerase database. Nucleic Acids Res..

[56] Jiang J., Wang Y., Sušac L., Chan H., Basu R., Zhou Z.H., Feigon J. (2018). Structure of telomerase with telomeric DNA. Cell.

[57] Chu T.W., D'Souza Y., Autexier C. (2016). The insertion in fingers domain in human telomerase can mediate enzyme processivity and telomerase recruitment to telomeres in a TPP1-dependent manner. Mol. Cell. Biol..

[58] Tsakiri K.D., Cronkhite J.T., Kuan P.J., Xing C., Raghu G., Weissler J.C., Rosenblatt R.L., Shay J.W., Garcia C.K. (2007). Adult-onset pulmonary fibrosis caused by mutations in telomerase. Proc. Natl. Acad. Sci. U. S. A..

[59] Vulliamy T., Marrone A., Goldman F., Dearlove A., Bessler M., Mason P.J., Dokal I. (2001). The RNA component of telomerase is mutated in autosomal dominant dyskeratosis congenita. Nature.

[60] Yamaguchi H., Baerlocher G.M., Lansdorp P.M., Chanock S.J., Nunez O., Sloand E., Young N.S. (2003). Mutations of the human telomerase RNA gene (TERC) in aplastic anemia and myelodysplastic syndrome. Blood.

[61] Lai C.K., Miller M.C., Collins K. (2003). Roles for RNA in telomerase nucleotide and repeat addition processivity. Mol. Cell.

[62] Fu D., Collins K. (2003). Distinct biogenesis pathways for human telomerase RNA and H/ACA small nucleolar RNAs. Mol. Cell.

[63] Errington T.M., Fu D., Wong J.M., Collins K. (2008). Disease-associated human telomerase RNA variants show loss of function for telomere synthesis without dominant-negative interference. Mol. Cell. Biol..

[64] Cerone M.A., Ward R.J., Londoño-Vallejo J.A., Autexier C. (2005). Telomerase RNA mutated in autosomal dyskeratosis congenita reconstitutes a weakly active telomerase enzyme defective in telomere elongation. Cell Cycle.

[65] Robart A.R., Collins K. (2010). Investigation of human telomerase holoenzyme assembly, activity, and processivity using disease-linked subunit variants. J. Biol. Chem..

[66] Theimer C.A., Finger L.D., Trantirek L., Feigon J. (2003). Mutations linked to dyskeratosis congenita cause changes in the structural equilibrium in telomerase RNA. Proc. Natl. Acad. Sci. U. S. A..

[67] Theimer C.A., Finger L.D., Feigon J. (2003). YNMG tetraloop formation by a dyskeratosis congenita mutation in human telomerase RNA. RNA.

[68] Marrone A., Stevens D., Vulliamy T., Dokal I., Mason P.J. (2004). Heterozygous telomerase RNA mutations found in dyskeratosis congenita and aplastic anemia reduce telomerase activity via haploinsufficiency. Blood.

[69] Trahan C., Dragon F. (2009). Dyskeratosis congenita mutations in the H/ACA domain of human telomerase RNA affect its assembly into a pre-RNP. RNA.

[70] Mitchell J.R., Collins K. (2000). Human telomerase activation requires two independent interactions between telomerase RNA and telomerase reverse transcriptase. Mol. Cell.

[71] Matera A.G., Terns R.M., Terns M.P. (2007). Non-coding RNAs: lessons from the small nuclear and small nucleolar RNAs. Nat. Rev. Mol. Cell Biol..

[72] Mitchell J.R., Cheng J., Collins K. (1999). A box H/ACA small nucleolar RNA-like domain at the human telomerase RNA 3' end. Mol. Cell. Biol..

[73] Vulliamy T.J., Knight S.W., Dokal I., Mason P.J. (1997). Skewed X-inactivation in carriers of X-linked dyskeratosis congenita. Blood.

[74] Xu J., Khincha P.P., Giri N., Alter B.P., Savage S.A., Wong J.M. (2016). Investigation of chromosome X inactivation and clinical phenotypes in female carriers of DKC1 mutations. Am. J. Hematol..

[75] Rashid R., Liang B., Baker D.L., Youssef O.A., He Y., Phipps K., Terns R.M., Terns M.P., Li H. (2006). Crystal structure of a Cbf5-Nop10-Gar1 complex and implications in RNA-guided pseudouridylation and dyskeratosis congenita. Mol. Cell.

[76] Li L., Ye K. (2006). Crystal structure of an H/ACA box ribonucleoprotein particle. Nature.

[77] Trahan C., Martel C., Dragon F. (2010). Effects of dyskeratosis congenita mutations in dyskerin, NHP2 and NOP10 on assembly of H/ACA pre-RNPs. Hum. Mol. Genet..

[78] Wong J.M., Collins K. (2006). Telomerase RNA level limits telomere maintenance in X-linked dyskeratosis congenita. Genes Dev..

[79] Zeng X.L., Thumati N.R., Fleisig H.B., Hukezalie K.R., Savage S.A., Giri N., Alter B.P., Wong J.M. (2012). The accumulation and not the specific activity of telomerase ribonucleoprotein determines telomere maintenance deficiency in X-linked dyskeratosis congenita. Hum. Mol. Genet..

[80] MacNeil D.E., Lambert-Lanteigne P., Autexier C. (2019). N-terminal residues of human dyskerin are required for interactions with telomerase RNA that prevent RNA degradation. Nucleic Acids Res..

[81] Walne A.J., Vulliamy T., Marrone A., Beswick R., Kirwan M., Masunari Y., Al-Qurashi F.H., Aljurf M., Dokal I. (2007). Genetic heterogeneity in autosomal recessive dyskeratosis congenita with one subtype due to mutations in the telomerase-associated protein NOP10. Hum. Mol. Genet..

[82] Vulliamy T., Beswick R., Kirwan M., Marrone A., Digweed M., Walne A., Dokal I. (2008). Mutations in the telomerase component NHP2 cause the premature ageing syndrome dyskeratosis congenita. Proc. Natl. Acad. Sci. U. S. A..

[83] Stuart B.D., Choi J., Zaidi S., Xing C., Holohan B., Chen R., Choi M., Dharwadkar P., Torres F., Girod C.E., Weissler J., Fitzgerald J., Kershaw C., Klesney-Tait J., Mageto Y. (2015). Exome sequencing links mutations in PARN and RTEL1 with familial pulmonary fibrosis and telomere shortening. Nat. Genet..

[84] Moon D.H., Segal M., Boyraz B., Guinan E., Hofmann I., Cahan P., Tai A.K., Agarwal S. (2015). Poly(A)-specific ribonuclease (PARN) mediates 3'-end maturation of the telomerase RNA component. Nat. Genet..

[85] Tummala H., Walne A., Collopy L., Cardoso S., de la Fuente J., Lawson S., Powell J., Cooper N., Foster A., Mohammed S., Plagnol V., Vulliamy T., Dokal I. (2015). Poly(A)-specific ribonuclease deficiency impacts telomere biology and causes dyskeratosis congenita. J. Clin. Invest..

[86] Shukla S., Schmidt J.C., Goldfarb K.C., Cech T.R., Parker R. (2016). Inhibition of telomerase RNA decay rescues telomerase deficiency caused by dyskerin or PARN defects. Nat. Struct. Mol. Biol..

[87] Boyraz B., Moon D.H., Segal M., Muosieyiri M.Z., Aykanat A., Tai A.K., Cahan P., Agarwal S. (2016). Posttranscriptional manipulation of TERC reverses molecular hallmarks of telomere disease. J. Clin. Invest..

[88] Fok W.C., Shukla S., Vessoni A.T., Brenner K.A., Parker R., Sturgeon C.M., Batista L.F.Z. (2019). Posttranscriptional modulation of TERC by PAPD5 inhibition rescues hematopoietic development in dyskeratosis congenita. Blood.

[89] Tseng C.K., Wang H.F., Burns A.M., Schroeder M.R., Gaspari M., Baumann P. (2015). Human telomerase RNA processing and quality control. Cell Rep..

[90] Nguyen D., Grenier St-Sauveur V., Bergeron D., Dupuis-Sandoval F., Scott M.S., Bachand F. (2015). A polyadenylation-dependent 3' end maturation pathway is required for the synthesis of the human telomerase RNA. Cell Rep..

[91] Roake C.M., Chen L., Chakravarthy A.L., Ferrell J.E., Raffa G.D., Artandi S.E. (2019). Disruption of telomerase RNA maturation kinetics precipitates disease. Mol. Cell.

[92] Nagpal N., Wang J., Zeng J., Lo E., Moon D.H., Luk K., Braun R.O., Burroughs L.M., Keel S.B., Reilly C., Lindsley R.C., Wolfe S.A., Tai A.K., Cahan P., Bauer D.E. (2020). Small-molecule PAPD5 inhibitors restore telomerase activity in patient stem cells. Cell Stem Cell.

[93] Mueller H., Lopez A., Tropberger P., Wildum S., Schmaler J., Pedersen L., Han X., Wang Y., Ottosen S., Yang S., Young J.A.T., Javanbakht H. (2019). PAPD5/7 are host factors that are required for hepatitis B virus RNA stabilization. Hepatology.

[94] Shukla S., Jeong H.C., Sturgeon C.M., Parker R., Batista L.F.Z. (2020). Chemical inhibition of PAPD5/7 rescues telomerase function and hematopoiesis in dyskeratosis congenita. Blood Adv..

[95] Gable D.L., Gaysinskaya V., Atik C.C., Talbot C.C., Kang B., Stanley S.E., Pugh E.W., Amat-Codina N., Schenk K.M., Arcasoy M.O., Brayton C., Florea L., Armanios M. (2019). The nuclear exosome targeting component, is mutated in familial pulmonary fibrosis and is required for telomerase RNA maturation. Genes Dev..

[96] Kilchert C., Wittmann S., Vasiljeva L. (2016). The regulation and functions of the nuclear RNA exosome complex. Nat. Rev. Mol. Cell Biol..

[97] Venteicher A.S., Abreu E.B., Meng Z., McCann K.E., Terns R.M., Veenstra T.D., Terns M.P., Artandi S.E. (2009). A human telomerase holoenzyme protein required for cajal body localization and telomere synthesis. Science.

[98] Tycowski K.T., Shu M.D., Kukoyi A., Steitz J.A. (2009). A conserved WD40 protein binds the cajal body localization signal of scaRNP particles. Mol. Cell.

[99] Roake C.M., Artandi S.E. (2020). Regulation of human telomerase in homeostasis and disease. Nat. Rev. Mol. Cell Biol..

[100] Zhong F., Savage S.A., Shkreli M., Giri N., Jessop L., Myers T., Chen R., Alter B.P., Artandi S.E. (2011). Disruption of telomerase trafficking by TCAB1 mutation causes dyskeratosis congenita. Genes Dev..

[101] Batista L.F., Pech M.F., Zhong F.L., Nguyen H.N., Xie K.T., Zaug A.J., Crary S.M., Choi J., Sebastiano V., Cherry A., Giri N., Wernig M., Alter B.P., Cech T.R., Savage S.A. (2011). Telomere shortening and loss of self-renewal in dyskeratosis congenita induced pluripotent stem cells. Nature.

[102] Freund A., Zhong F.L., Venteicher A.S., Meng Z., Veenstra T.D., Frydman J., Artandi S.E. (2014). Proteostatic control of telomerase function through TRiC-mediated folding of TCAB1. Cell.

[103] Chen L., Roake C.M., Freund A., Batista P.J., Tian S., Yin Y.A., Gajera C.R., Lin S., Lee B., Pech M.F., Venteicher A.S., Das R., Chang H.Y., Artandi S.E. (2018). An activity switch in human telomerase based on RNA conformation and shaped by TCAB1. Cell.

[104] de Lange T. (2018). Shelterin-mediated telomere protection. Annu. Rev. Genet..

[105] Grill S., Bisht K., Tesmer V.M., Shami A.N., Hammoud S.S., Nandakumar J. (2019). Two separation-of-function isoforms of human TPP1 dictate telomerase regulation in somatic and germ cells. Cell Rep..

[106] Grill S., Tesmer V.M., Nandakumar J. (2018). The N terminus of the OB domain of telomere protein TPP1 is critical for telomerase action. Cell Rep..

[107] Kocak H., Ballew B.J., Bisht K., Eggebeen R., Hicks B.D., Suman S., O'Neil A., Giri N., Maillard I., Alter B.P., Keegan C.E., Nandakumar J., Savage S.A., Laboratory N.D.C.G.R., Group N.D.C.S.W. (2014). Hoyeraal-Hreidarsson syndrome caused by a germline mutation in the TEL patch of the telomere protein TPP1. Genes Dev..

[108] Guo Y., Kartawinata M., Li J., Pickett H.A., Teo J., Kilo T., Barbaro P.M., Keating B., Chen Y., Tian L., Al-Odaib A., Reddel R.R., Christodoulou J., Xu X., Hakonarson H. (2014). Inherited bone marrow failure associated with germline mutation of ACD, the gene encoding telomere protein TPP1. Blood.

[109] Tummala H., Collopy L.C., Walne A.J., Ellison A., Cardoso S., Aksu T., Yarali N., Aslan D., Fikret Akata R., Teo J., Songyang Z., Pontikos N., Fitzgibbon J., Tomita K., Vulliamy T. (2018). Homozygous OB-fold variants in telomere protein TPP1 are associated with dyskeratosis congenita-like phenotypes. Blood.

[110] Hu C., Rai R., Huang C., Broton C., Long J., Xu Y., Xue J., Lei M., Chang S., Chen Y. (2017). Structural and functional analyses of the mammalian TIN2-TPP1-TRF2 telomeric complex. Cell Res..

[111] Takai K.K., Kibe T., Donigian J.R., Frescas D., de Lange T. (2011). Telomere protection by TPP1/POT1 requires tethering to TIN2. Mol. Cell.

[112] Frescas D., de Lange T. (2014). Binding of TPP1 protein to TIN2 protein is required for POT1a,b protein-mediated telomere protection. J. Biol. Chem..

[113] Chen Y., Yang Y., van Overbeek M., Donigian J.R., Baciu P., de Lange T., Lei M. (2008). A shared docking motif in TRF1 and TRF2 used for differential recruitment of telomeric proteins. Science.

[114] Kaminker P.G., Kim S.H., Desprez P.Y., Campisi J. (2009). A novel form of the telomere-associated protein TIN2 localizes to the nuclear matrix. Cell Cycle.

[115] Pike A.M., Strong M.A., Ouyang J.P.T., Greider C.W. (2019). TIN2 functions with TPP1/POT1 to stimulate telomerase processivity. Mol. Cell. Biol..

[116] Nelson N.D., Dodson L.M., Escudero L., Sukumar A.T., Williams C.L., Mihalek I., Baldan A., Baird D.M., Bertuch A.A. (2018). The C-terminal extension unique to the long isoform of the shelterin component TIN2 enhances its interaction with TRF2 in a phosphorylation- and dyskeratosis congenita cluster-dependent fashion. Mol. Cell. Biol..

[117] Lim C.J., Zaug A.J., Kim H.J., Cech T.R. (2017). Reconstitution of human shelterin complexes reveals unexpected stoichiometry and dual pathways to enhance telomerase processivity. Nat. Commun..

[118] Bisht K., Smith E.M., Tesmer V.M., Nandakumar J. (2016). Structural and functional consequences of a disease mutation in the telomere protein TPP1. Proc. Natl. Acad. Sci. U. S. A..

[119] Walne A.J., Vulliamy T., Beswick R., Kirwan M., Dokal I. (2008). TINF2 mutations result in very short telomeres: analysis of a large cohort of patients with dyskeratosis congenita and related bone marrow failure syndromes. Blood.

[120] Savage S.A., Giri N., Baerlocher G.M., Orr N., Lansdorp P.M., Alter B.P. (2008). TINF2, a component of the shelterin telomere protection complex, is mutated in dyskeratosis congenita. Am. J. Hum. Genet..

[121] Frescas D., de Lange T. (2014). A TIN2 dyskeratosis congenita mutation causes telomerase-independent telomere shortening in mice. Genes Dev..

[122] Frank A.K., Tran D.C., Qu R.W., Stohr B.A., Segal D.J., Xu L. (2015). The shelterin TIN2 subunit mediates recruitment of telomerase to telomeres. PLoS Genet..

[123] Xin Z.T., Ly H. (2012). Characterization of interactions between naturally mutated forms of the TIN2 protein and its known protein partners of the shelterin complex. Clin. Genet..

[124] Yang D., He Q., Kim H., Ma W., Songyang Z. (2011). TIN2 protein dyskeratosis congenita missense mutants are defective in association with telomerase. J. Biol. Chem..

[125] Canudas S., Houghtaling B.R., Bhanot M., Sasa G., Savage S.A., Bertuch A.A., Smith S. (2011). A role for heterochromatin protein 1gamma at human telomeres. Genes Dev..

[126] Alder J.K., Stanley S.E., Wagner C.L., Hamilton M., Hanumanthu V.S., Armanios M. (2015). Exome sequencing identifies mutant TINF2 in a family with pulmonary fibrosis. Chest.

[127] Fukuhara A., Tanino Y., Ishii T., Inokoshi Y., Saito K., Fukuhara N., Sato S., Saito J., Ishida T., Yamaguchi H., Munakata M. (2013). Pulmonary fibrosis in dyskeratosis congenita with TINF2 gene mutation. Eur. Respir. J..

[128] Kannengiesser C., Borie R., Revy P. (2014). Pulmonary fibrosis associated with TINF2 gene mutation: is somatic reversion required?. Eur. Respir. J..

[129] Vannier J.B., Sarek G., Boulton S.J. (2014). RTEL1: functions of a disease-associated helicase. Trends Cell Biol..

[130] Vannier J.B., Sandhu S., Petalcorin M.I., Wu X., Nabi Z., Ding H., Boulton S.J. (2013). RTEL1 is a replisome-associated helicase that promotes telomere and genome-wide replication. Science.

[131] Vannier J.B., Pavicic-Kaltenbrunner V., Petalcorin M.I., Ding H., Boulton S.J. (2012). RTEL1 dismantles T loops and counteracts telomeric G4-DNA to maintain telomere integrity. Cell.

[132] Sarek G., Vannier J.B., Panier S., Petrini J.H., Boulton S.J. (2015). TRF2 recruits RTEL1 to telomeres in S phase to promote t-loop unwinding. Mol. Cell.

[133] Sarek G., Kotsantis P., Ruis P., Van Ly D., Margalef P., Borel V., Zheng X.F., Flynn H.R., Snijders A.P., Chowdhury D., Cesare A.J., Boulton S.J. (2019). CDK phosphorylation of TRF2 controls t-loop dynamics during the cell cycle. Nature.

[134] Deng Z., Glousker G., Molczan A., Fox A.J., Lamm N., Dheekollu J., Weizman O.E., Schertzer M., Wang Z., Vladimirova O., Schug J., Aker M., Londoño-Vallejo A., Kaestner K.H., Lieberman P.M. (2013). Inherited mutations in the helicase RTEL1 cause telomere dysfunction and Hoyeraal-Hreidarsson syndrome. Proc. Natl. Acad. Sci. U. S. A..

[135] Ballew B.J., Joseph V., De S., Sarek G., Vannier J.B., Stracker T., Schrader K.A., Small T.N., O'Reilly R., Manschreck C., Harlan Fleischut M.M., Zhang L., Sullivan J., Stratton K., Yeager M. (2013). A recessive founder mutation in regulator of telomere elongation helicase 1, RTEL1, underlies severe immunodeficiency and features of Hoyeraal Hreidarsson syndrome. PLoS Genet..

[136] Ballew B.J., Yeager M., Jacobs K., Giri N., Boland J., Burdett L., Alter B.P., Savage S.A. (2013). Germline mutations of regulator of telomere elongation helicase 1, RTEL1, in dyskeratosis congenita. Hum. Genet..

[137] Walne A.J., Vulliamy T., Kirwan M., Plagnol V., Dokal I. (2013). Constitutional mutations in RTEL1 cause severe dyskeratosis congenita. Am. J. Hum. Genet..

[138] Le Guen T., Jullien L., Touzot F., Schertzer M., Gaillard L., Perderiset M., Carpentier W., Nitschke P., Picard C., Couillault G., Soulier J., Fischer A., Callebaut I., Jabado N., Londono-Vallejo A. (2013). Human RTEL1 deficiency causes Hoyeraal-Hreidarsson syndrome with short telomeres and genome instability. Hum. Mol. Genet..

[139] Porreca R.M., Glousker G., Awad A., Matilla Fernandez M.I., Gibaud A., Naucke C., Cohen S.B., Bryan T.M., Tzfati Y., Draskovic I., Londoño-Vallejo A. (2018). Human RTEL1 stabilizes long G-overhangs allowing telomerase-dependent over-extension. Nucleic Acids Res..

[140] Schertzer M., Jouravleva K., Perderiset M., Dingli F., Loew D., Le Guen T., Bardoni B., de Villartay J.P., Revy P., Londoño-Vallejo A. (2015). Human regulator of telomere elongation helicase 1 (RTEL1) is required for the nuclear and cytoplasmic trafficking of pre-U2 RNA. Nucleic Acids Res..

[141] Fedick A.M., Shi L., Jalas C., Treff N.R., Ekstein J., Kornreich R., Edelmann L., Mehta L., Savage S.A. (2015). Carrier screening of RTEL1 mutations in the Ashkenazi Jewish population. Clin. Genet..

[142] Sarek G., Vannier J.B., Panier S., Petrini J.H.J., Boulton S.J. (2016). TRF2 recruits RTEL1 to telomeres in S phase to promote T-loop unwinding. Mol. Cell.

[143] Chen L.Y., Redon S., Lingner J. (2012). The human CST complex is a terminator of telomerase activity. Nature.

[144] Miyake Y., Nakamura M., Nabetani A., Shimamura S., Tamura M., Yonehara S., Saito M., Ishikawa F. (2009). RPA-like mammalian Ctc1-Stn1-Ten1 complex binds to single-stranded DNA and protects telomeres independently of the Pot1 pathway. Mol. Cell.

[145] Surovtseva Y.V., Churikov D., Boltz K.A., Song X., Lamb J.C., Warrington R., Leehy K., Heacock M., Price C.M., Shippen D.E. (2009). Conserved telomere maintenance component 1 interacts with STN1 and maintains chromosome ends in higher eukaryotes. Mol. Cell.

[146] Wang F., Stewart J.A., Kasbek C., Zhao Y., Wright W.E., Price C.M. (2012). Human CST has independent functions during telomere duplex replication and C-strand fill-in. Cell Rep..

[147] Sun J., Yu E.Y., Yang Y., Confer L.A., Sun S.H., Wan K., Lue N.F., Lei M. (2009). Stn1-Ten1 is an Rpa2-Rpa3-like complex at telomeres. Genes Dev..

[148] Wu P., Takai H., de Lange T. (2012). Telomeric 3' overhangs derive from resection by Exo1 and Apollo and fill-in by POT1b-associated CST. Cell.

[149] Rice C., Skordalakes E. (2016). Structure and function of the telomeric CST complex. Comput. Struct. Biotechnol. J..

[150] Price C.M., Cech T.R. (1987). Telomeric DNA-protein interactions of oxytricha macronuclear DNA. Genes Dev..

[151] Zhang M., Wang B., Li T., Liu R., Xiao Y., Geng X., Li G., Liu Q., Price C.M., Liu Y., Wang F. (2019). Mammalian CST averts replication failure by preventing G-quadruplex accumulation. Nucleic Acids Res..

[152] Bhattacharjee A., Wang Y., Diao J., Price C.M. (2017). Dynamic DNA binding, junction recognition and G4 melting activity underlie the telomeric and genome-wide roles of human CST. Nucleic Acids Res..

[153] Chastain M., Zhou Q., Shiva O., Fadri-Moskwik M., Whitmore L., Jia P., Dai X., Huang C., Ye P., Chai W. (2016). Human CST facilitates genome-wide RAD51 recruitment to GC-rich repetitive sequences in response to replication stress. Cell Rep..

[154] Stewart J.A., Wang F., Chaiken M.F., Kasbek C., Chastain P.D., Wright W.E., Price C.M. (2012). Human CST promotes telomere duplex replication and general replication restart after fork stalling. EMBO J..

[155] Keller R.B., Gagne K.E., Usmani G.N., Asdourian G.K., Williams D.A., Hofmann I., Agarwal S. (2012). CTC1 Mutations in a patient with dyskeratosis congenita. Pediatr. Blood Cancer.

[156] Walne A.J., Bhagat T., Kirwan M., Gitiaux C., Desguerre I., Leonard N., Nogales E., Vulliamy T., Dokal I.S. (2013). Mutations in the telomere capping complex in bone marrow failure and related syndromes. Haematologica.

[157] Anderson B.H., Kasher P.R., Mayer J., Szynkiewicz M., Jenkinson E.M., Bhaskar S.S., Urquhart J.E., Daly S.B., Dickerson J.E., O'Sullivan J., Leibundgut E.O., Muter J., Abdel-Salem G.M., Babul-Hirji R., Baxter P. (2012). Mutations in CTC1, encoding conserved telomere maintenance component 1, cause Coats plus. Nat. Genet..

[158] Chen L.Y., Majerská J., Lingner J. (2013). Molecular basis of telomere syndrome caused by CTC1 mutations. Genes Dev..

[159] Gu P., Chang S. (2013). Functional characterization of human CTC1 mutations reveals novel mechanisms responsible for the pathogenesis of the telomere disease coats plus. Aging Cell.

[160] Gu P., Jia S., Takasugi T., Smith E., Nandakumar J., Hendrickson E., Chang S. (2018). CTC1-STN1 coordinates G- and C-strand synthesis to regulate telomere length. Aging Cell.

[161] Lim C.J., Barbour A.T., Zaug A.J., Goodrich K.J., McKay A.E., Wuttke D.S., Cech T.R. (2020). The structure of human CST reveals a decameric assembly bound to telomeric DNA. Science.

[162] Adam M.P., Ardinger H.H., Pagon R.A., Wallace S.E., Bean L.J.H., Stephens K., Amemiya A. (1993). GeneReviews.

[163] Rudolph K.L., Chang S., Lee H.W., Blasco M., Gottlieb G.J., Greider C., DePinho R.A. (1999). Longevity, stress response, and cancer in aging telomerase-deficient mice. Cell.

[164] Jones M., Osawa G., Regal J.A., Weinberg D.N., Taggart J., Kocak H., Friedman A., Ferguson D.O., Keegan C.E., Maillard I. (2014). Hematopoietic stem cells are acutely sensitive to Acd shelterin gene inactivation. J. Clin. Invest..

[165] Agarwal S., Loh Y.H., McLoughlin E.M., Huang J., Park I.H., Miller J.D., Huo H., Okuka M., Dos Reis R.M., Loewer S., Ng H.H., Keefe D.L., Goldman F.D., Klingelhutz A.J., Liu L. (2010). Telomere elongation in induced pluripotent stem cells from dyskeratosis congenita patients. Nature.

